# A pharmacokinetic model including arrival time for two inputs and compensating for varying applied flip-angle in dynamic gadoxetic acid-enhanced MR imaging

**DOI:** 10.1371/journal.pone.0220835

**Published:** 2019-08-15

**Authors:** Tian Zhang, Jurgen H. Runge, Cristina Lavini, Jaap Stoker, Thomas van Gulik, Kasia P. Cieslak, Lucas J. van Vliet, Frans M. Vos

**Affiliations:** 1 Department of Imaging Physics, Delft University of Technology, Delft, The Netherlands; 2 Department of Radiology, Academic Medical Center, Amsterdam, The Netherlands; 3 Department of Surgery, Academic Medical Center, Amsterdam, The Netherlands; University of Chicago, UNITED STATES

## Abstract

**Purpose:**

Pharmacokinetic models facilitate assessment of properties of the micro-vascularization based on DCE-MRI data. However, accurate pharmacokinetic modeling in the liver is challenging since it has two vascular inputs and it is subject to large deformation and displacement due to respiration.

**Methods:**

We propose an improved pharmacokinetic model for the liver that (1) analytically models the arrival-time of the contrast agent for both inputs separately; (2) implicitly compensates for signal fluctuations that can be modeled by varying applied flip-angle e.g. due to B1-inhomogeneity.

Orton’s AIF model is used to analytically represent the vascular input functions. The inputs are independently embedded into the Sourbron model. B1-inhomogeneity-driven variations of flip-angles are accounted for to justify the voxel’s displacement with respect to a pre-contrast image.

**Results:**

The new model was shown to yield lower root mean square error (RMSE) after fitting the model to all but a minority of voxels compared to Sourbron’s approach. Furthermore, it outperformed this existing model in the majority of voxels according to three model-selection criteria.

**Conclusion:**

Our work primarily targeted to improve pharmacokinetic modeling for DCE-MRI of the liver. However, other types of pharmacokinetic models may also benefit from our approaches, since the techniques are generally applicable.

## Introduction

Dynamic Contrast-Enhanced MRI (DCE-MRI) is a technique that can be applied to assess properties of the micro-vascularization in organs such as the liver, breast, and kidney [1][2]. Pharmacokinetic (PK) modeling in the liver is more challenging than in the rest of the body since the liver has two vascular inputs: the hepatic artery and the portal vein. Furthermore, contrary to standard Gd-based contrast media, the hepatobiliary contrast agent Gadoxetate disodium (Primovist^TM^, Bayer pharmaceutical) is also taken up by the hepatocytes. As such an additional compartment should be taken into account in a pharmacokinetic model. Finally, the uptake rate of the hepatocytes is low and for this reason DCE-MRI may take up to 20 minutes or more [1]. During image acquisition the liver can experience large deformations and displacements, which may significantly influence the signal intensity (e.g. due to B1-inhomogeneity). These issues result in the fact that accurate pharmacokinetic modeling in the liver is far from trivial.

### Related work

Quantitative analysis of liver function with MRI using Gd-EOB-DTPA in rabbits was first proposed by Ryeom *et al*. [3] in 2004. Using a deconvolution technique, the estimated hepatic extraction fraction (HEF) showed correlation with liver function measured through the plasma’s retention rate after indocyane green injection. Subsequently, Nilsson *et al*. [4] applied the same liver model to humans with a more efficient deconvolution technique called truncated singular value decomposition (TSVD). However, this deconvolution approach regarded the hepatic artery as the sole input, and ignored the portal vein. A dual-input one-compartmental model was already proposed in 2002, but this model focused on extracellular contrast agents such as Gd-DTPA (Magnevist, Bayer Schering Pharma, Berlin, Germany) [5]. By adding an intracellular compartment, Sourbron *et al*. [2] created a dual-input, two-compartmental model that accounted for Gd-EOB-DTPA metabolization by the hepatic cells in 2012. A limitation of Sourbron’s model is that it ignores the extraction rate of hepatocytes, i.e. the efflux to the bile canaliculi. To solve this, Ulloa *et al*. [6] and Forsgren *et al*. [7] modeled the transport of the contrast agent from the hepatocytes to the bile via nonlinear Michaelis-Menten kinetics in rats and humans respectively. Georgiou *et al*. [8] tried to simplify the efflux transport by a simple linear approximation. Recently, Ning *et al*. [9] correlated pharmacokinetic parameters estimated from different models with a blood chemistry test. It was found that the relative liver uptake rate estimated from the model without bile efflux transport significantly correlated with direct bilirubin (*r* = -0.52, *p* = 0.015), prealbumin (*r* = 0.58, *p* = 0.015) and prothrombin time (*r* = -0.51, *p* = 0.026). Furthermore, only insignificant correlations were found using the model with efflux transport. Accordingly, our work regards Sourbron’s model [2] as the starting point, i.e. opting for a model without bile efflux transport.

The Arterial Input Function (AIF) represents the time-dependent arterial contrast agent concentration, that is typically used in pharmacokinetic modelling of dynamic imaging data. Population-averaged parametrized models (e.g. (Orton, Parker) have been used as such. The AIF model described by Orton et al. [10] parametrizes the AIF as a sum of two functions, one describing the first passage of the bolus peak, and the other representing the wash-out of CA in the tail of the AIF. Alternatively, Parker’s model [11] can describe the second pass (recirculation) of the contrast agent. However, the latter feature may not always be visible in the MRI data, e.g. due to low temporal resolution (see e.g.[12]).

In Sourbron’s approach, the delay of the arterial input is empirically determined by the best model fit over a discrete set of values. This might limit the accuracy of the PKM parameter estimation and could restrict its applicability. Furthermore, the method does not take the effects of liver motion on the signal intensity into account. Such motion not only causes misalignment, which should be compensated for using image registration, but it may also induce other signal fluctuation, due to motion-induced time-varying B1-inhomogeneity caused by the movement of the bowel in the field of view [13].

Previously, several papers investigated the influence of B1-inhomogeneity on pharmacokinetic modeling. For example, Park *et al*. [14] and Sengupta *et al* [15] conducted a simulation and an experimental study respectively showing that a small degree of B1-inhomogeneity can cause a significant error in the estimated PKM parameters. Gach *et al*. [16] corrected the B1 inhomogeneity by performing a 3D GRE sequence with various flip angles (2–30°) in phantoms to obtain standards for normalizing the 3D GRE DCE MR images. Alternatively, Van Schie *et al*. [17] combined variable flip angle (VFA) and Look-Locker (LL) sequences to obtain a B1-inhomogeneity map for DCE imaging. Such a B1-map may also be obtained by means of the DREAM sequence [18]. Essentially, all these methods attempt to correct the B1-inhomogeneity based on auxiliary sequences. However, this not only makes the imaging even more time-consuming, it conventionally yields static B1-maps whereas fluctuations due to motion remain hard to account for.

## Objective

In this paper we aim to improve pharmacokinetic modeling of liver DCE MRI data. Therefore, two novelties are introduced in the PK modeling. First, the arterial input function proposed by Orton is integrated into Sourbron’s PK model. This enables that the arrival times of contrast from the portal vein and the hepatic artery are separately included in the model and estimated simultaneously with the PK model parameters. Secondly, the deformation and displacement of the liver is estimated and used to correct for changes in signal intensity such as the ones caused by B1-inhomogeneities.The effectiveness of the new model will be assessed by several experiments.

## Materials and methods

### Data acquisition

Patients diagnosed with one or more liver lesions and who were scheduled for ^99m^Tc-mebrofenin HBS as part of the preoperative workup were included in this prospective pilot study. Patients with general contraindications for MRI, chronic renal insufficiency, known or family history of congenital prolonged QT-syndrome, current use of cardiac repolarization time prolonging drugs (such as class 3 anti-arrhythmic drugs), history of arrhythmia after the use of cardiac repolarization time prolonging drugs and history of allergic reaction to gadolinium-containing compounds were excluded from participation. 11 subjects were included for this research project. Subjects’ characteristics can be seen in Table 1.

**Table 1:** Subjects’ characteristics.

Characteristics n	11
Age, median (IQR)	64 (58–67)
Male sex, n (%)	6 (55%)
BMI, kg/m^2^, median (IQR)	22 (21.7–27.3)
BSA, m^2^, median (IQR)	1.8 (1.7–2.1)
Diagnosis, n (%)	Colorectal liver metastasis: 5 (46%)
	Hepatocellular carcinoma: 2 (18%)
	Benign: 4 (36%)
Neo-adjuvant chemotherapy, n (%)	3 (27%)
Preoperative biliary drainage, n (%)	1 (9%)

The study was approved by the ethical review board of the Amsterdam University Medical Centers and registered under ID NL45755.018.13. Written informed consent was obtained from all individual participants included in the study.

DCE-MRI data were acquired coronally on a 3T Philips scanner at the AMC by means of a 3D T1-weighted Spoiled Gradient Echo sequence. The acquisition parameter settings were TE/TR = 2.30/3.75 ms, FA = 15°, matrix size = 128×128×44, voxel size = 3×3×5 mm^3^, acquisition time = 2.141 s for each volume; sampling interval (between images) was 2.141 s for volumes 1–81, 30 s for volumes 82–98, and 60 s for volumes 99–108. The total imaging time was approximately 20 minutes. Volumes 1–19 were acquired in the pre-contrast stage. Subjects held their breath during the acquisition of volumes 13–22, 33–42, 61–70 and 79–108.

In addition, dual refocusing echo acquisition mode (DREAM) images [18] were acquired to quantify the extent of the B1-inhomogeneity before the DCE sequence was acquired. The acquisition parameter settings were matrix size = 64×64×30, voxel size = 8.28×8.28×8.80 mm^3^, nominal STEAM flip-angle α = 60°, nominal imaging flip-angle*β* = 10°, TE_STE_ = 1.06 ms, TE_FID_ = 2.30 ms, TR = 3.84 ms. Essentially, the DREAM sequence produces a map in which the value of every voxel represents the ratio between the real flip-angle and the programmed flip-angle. We will refer to it as the ‘zeta’ map.

### Image registration and liver segmentation

Image registration is required to achieve spatial correspondence between voxels of the DCE-MRI data prior to PK modeling. In this work, each 4D DCE-MR dynamic is registered to the last dynamic volume. In order to do so, we apply the Modality Independent Neighborhood Descriptor (MIND) method [19], which is a state-of-the-art technique for multi-modal image registration. Essentially, it relies on a patch-based descriptor of the structure in a local neighborhood

$$MIND\left(I,x,r\right)=\frac{1}{n}\exp\left(-\frac{D_{p}\left(I,x,x+r\right)}{V\left(I,x\right)}\right),$$ (1)

in which *I* is an image, **r** an offset in neighborhood *P* of size *R*×*R*×*R* around position **x** and *n* a normalization constant; *D*_p_ is the distance between two image patches, measured by the sum of squared differences (SSD):

$$D_{p}\left(I,x_{1},x_{2}\right)=\sum_{p\in P}{\left(I\left(x_{1}+p\right)-I\left(x_{2}+p\right)\right)}^{2}.$$ (2)

where *V(I*, **x**) is the mean of the patch distances in a small neighborhood *N*

$$V\left(I,x\right)=\frac{1}{num\left(N\right)}\sum_{p\in P}D_{p}\left(I,x,x+n\right).$$ (3)

The MIND registration can be described as

$$u^{*}=\underset{u}{\arg\;\min}\left\{\sum_{x}\left[\frac{1}{\left|R\right|}\sum_{r\in R}\left|MIND\left(I,x,r\right)-MIND\left(J,x,r\right)\right|\right]+\alpha {\left|\nabla u\left(x\right)\right|}^{2}\right\},$$ (4)

where **u** = (*u*, *v*, *w*) is the deformation field and *α* a coefficient that weighs a regularization term. Thus, the MIND registration method produces a 3D voxelwise, regularized deformation field. In this paper we follow the default setup from [19]: *R* = 3, *N* = *N*_6_ i.e. a six-connected neighborhood, patch size *D* = 3, and the regularization coefficient α = 0.1.

Furthermore, we segment the liver, defining our region of interest. As we apply a liver-specific contrast agent, the surrounding organs show less signal enhancement than the liver. Maximal image contrast is achieved by subtracting the first dynamic of the series from the last, after registration. Subsequently, the liver is segmented based on the resulting “contrast” volume by means of a level set approach, which takes boundary as well as region information into consideration [20]. More implementation details can be found in [21]. We refrained from performing the segmentation in an anatomical scan, which indeed has higher resolution, but inferior contrast compared to the DCE MRI difference image.

**Fig 1:**
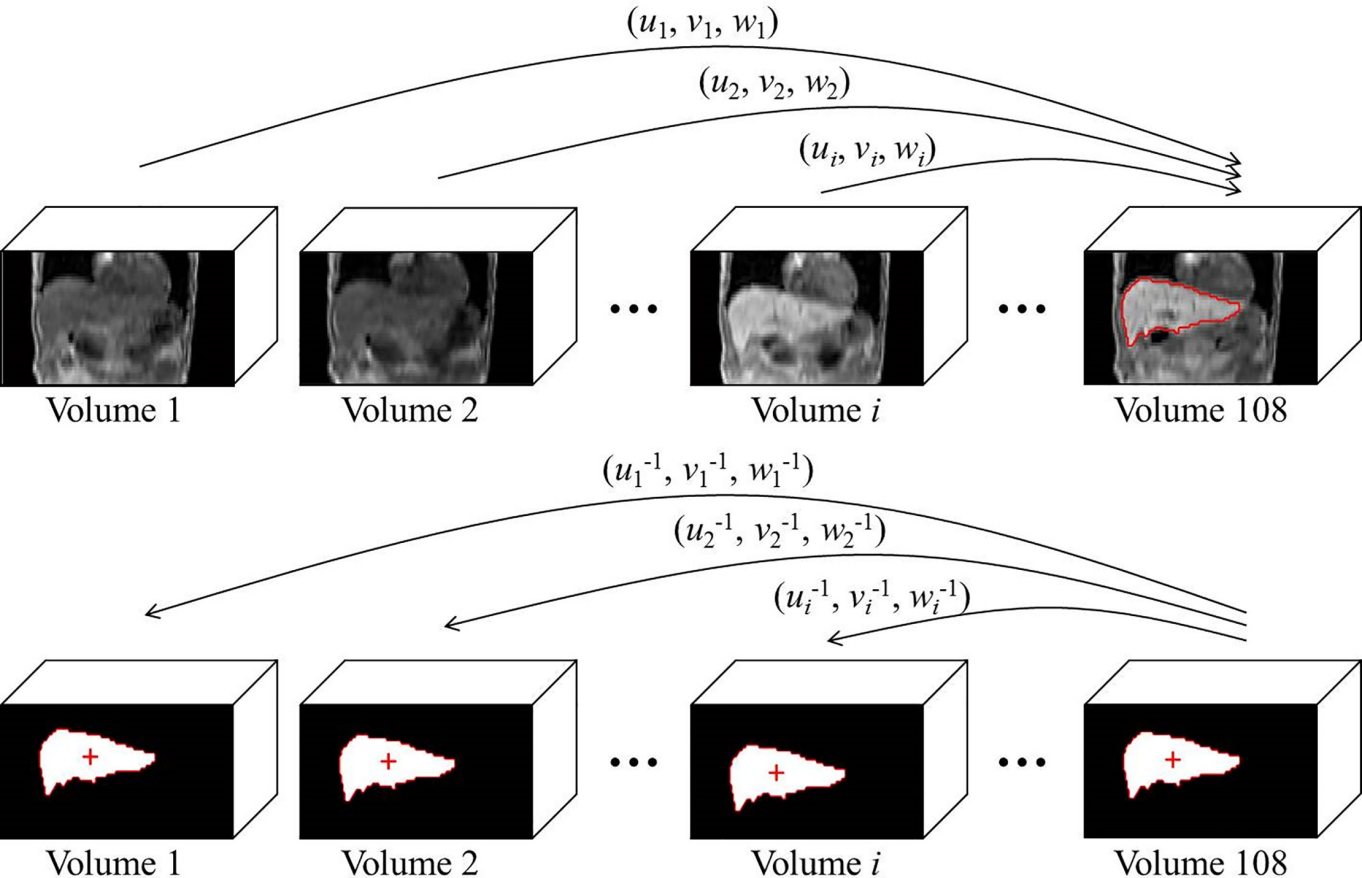
Diagram of showing how the inverse deformation field is used to warp the liver mask obtained in the fixed image to each moving image.

The obtained mask coarsely segments the liver across the registered DCE series. Simultaneously, inverting the registration transformations and applying them to the liver mask yield liver segmentations in each original dynamic, the transformations were performed as shown in Fig 1. Finally, we subtract from each deformation field the deformation field resulting from the registration of the first image to the last one. We do this merely for practical reasons, so that all deformation fields are relative to the first image in the series.

**Fig 2:**
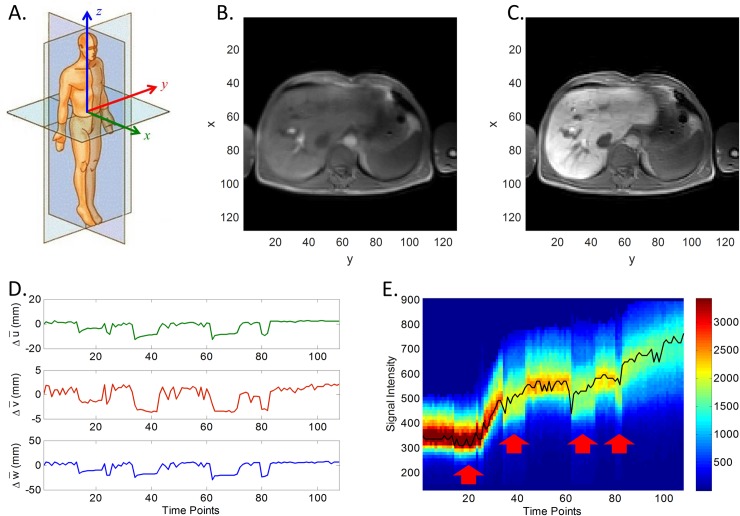
Illustration of our datasets. (A) Image coordinates; (B) and (C) are exemplary registered MR images in pre- and post-contrast stages, respectively; (D) Liver displacement curves in *x*, *y* and *z* directions; (E) The distribution of time intensity curves (TICs) for all liver voxels. The black line is the mode. NB: observe that the time interval between volumes was irregular (as described in section Data acquisition): 2.141 s for volumes 1–81, 30 s for volumes 82–98, and 60 s for volumes 99–108.

The liver’s mean relative displacement in a dynamic volume with respect to the first image is estimated as the distance between the liver mask’s center of mass and the deformed liver mask’s center of mass (in 3D), see Fig 2(A) and 2(D). The large displacements in some parts of the graph represent strong inhalation emanating from the breath holds (during dynamics 13–22, 33–42, 61–70 and 79–108). Notice that, at the same time, these large displacements coincide with abrupt offsets in the time intensity curves: see the arrows in Fig 2(E).

In the section *Varying effective flip-angle compensation* we will show how the liver displacements can be used to compensate for these intensity offsets.

In the following, the *x*-axis of the data corresponds to the anterior-posterior direction, the *y*-axis to the left-right direction and the *z*-axis to the superior-inferior direction, as show in Fig 2(A). Exemplary registered MR images in pre- and post-contrast stages are shown in Fig 2(B) and 2(C), respectively.

### Input function models

An arterial input function (AIF) represents the time-dependent arterial contrast agent (CA) concentration, that is used in PK modeling of dynamic imaging data. The AIF is often computed directly from the signal measured in an artery close to the tissue of interest. The liver, however, has two inputs: the hepatic artery’s AIF and the portal vein’s venous input function (VIF).

We assume that the profile of both input functions follows a slightly modified input function model described by Orton *et al*. [10]. This model parametrizes an input function as a sum of two functions, one describing the first passage of the bolus peak, and the other describing the wash-out of CA in the tail of the input function [22].

The bolus peak *C*_*B*_(*t*) is described by:

$$C_{B}\left(t\right)=a_{B}\mu_{B}^{2}e^{-\mu_{B}t}u\left(t\right),$$ (5)

with *u*(t) the unit step function. This function has been modified slightly with respect to the one described by Orton *et al*., such that the area under the curve of *C*_*B*_(*t*) is given by the parameter *a*_*B*_, while *μ*_*B*_ only affects the decay rate.

The tail of the AIF and VIF is expressed as a convolution between the bolus peak and a body transfer function G(*t*), which is modeled as

$$G\left(t\right)=a_{G}e^{-\mu_{G}t}u\left(t\right),$$ (6)

In which *a*_*G*_ determines the starting level of this decay function and *μ*_*G*_ governs the decay rate, which may reflect kidney functioning [10].

Thus, the complete input function is given by:

$$\begin{array}{l} C_{I}\left(t\right)=C_{B}\left(t\right)+C_{B}\left(t\right)*G\left(t\right)\\ \;=\left[A_{B}te^{-\mu_{B}t}+A_{G}\left(e^{-\mu_{G}t}-e^{-\mu_{B}t}\right)\right]u\left(t\right), \end{array}$$ (7)

with

$$\left\{ \begin{array}{l} A_{B}=a_{B}\mu_{B}^{2}\left(1-\frac{a_{G}}{\mu_{B}-\mu_{G}}\right)\\ A_{G}=\frac{a_{B}a_{G}\mu_{B}^{2}}{{\left(\mu_{B}-\mu_{G}\right)}^{2}} \end{array}\right.,$$

can be used to represent either the AIF or the VIF.

The liver’s AIF and VIF were estimated by semi-automatically segmenting a homogeneous region in the aorta and the portal vein, respectively [21]. The aorta and portal vein were segmented much in the same way as we performed the liver segmentation. Specifically, they were segmentedfrom volumes obtained by subtracting the first volume from the one in which maximal signal was measured in the aorta and portal vein, respectively. This was measurement was made in small, manually indicationed regions of interest in the aorta and portal vein.Subsequently, a level set segmentation algorithm [20] was applied to segment these structures.volumes. Finally, the resulting segmentations were eroded by 3x3 kernel, to remove partial volume voxels.

Subsequently, the top three of the most enhancing time intensity curves of the voxels in both regions were separately averaged and converted into time concentration curves (TCC) assuming a nonlinear relationship between signal intensity and concentration of contrast agent [23]. Finally, the input function parameters were estimated by fitting Orton’s model to these data. These fits yield different parameters for AIF and VIF.

An advantage of our approach is that noise on the input function is suppressed, because a smooth, parameterized representation is fit to the data. However, not all features contained in the original data may be represented, especially a second pass of the bolus peak, which is not contained in Orton’s model. We considered this limitation acceptable as, we could not visually identify a second peak corresponding to a second bolus pass in the hepatic artery let alone in the portal vein for any data set.

Furthermore, the parameterized input functions can be analytically integrated in our PK model (see below). As such, it allows for a continuous estimate of the time delay with which the AIF and VIF arrive in a voxel under investigation.

### Sourbron’s model

**Fig 3:**
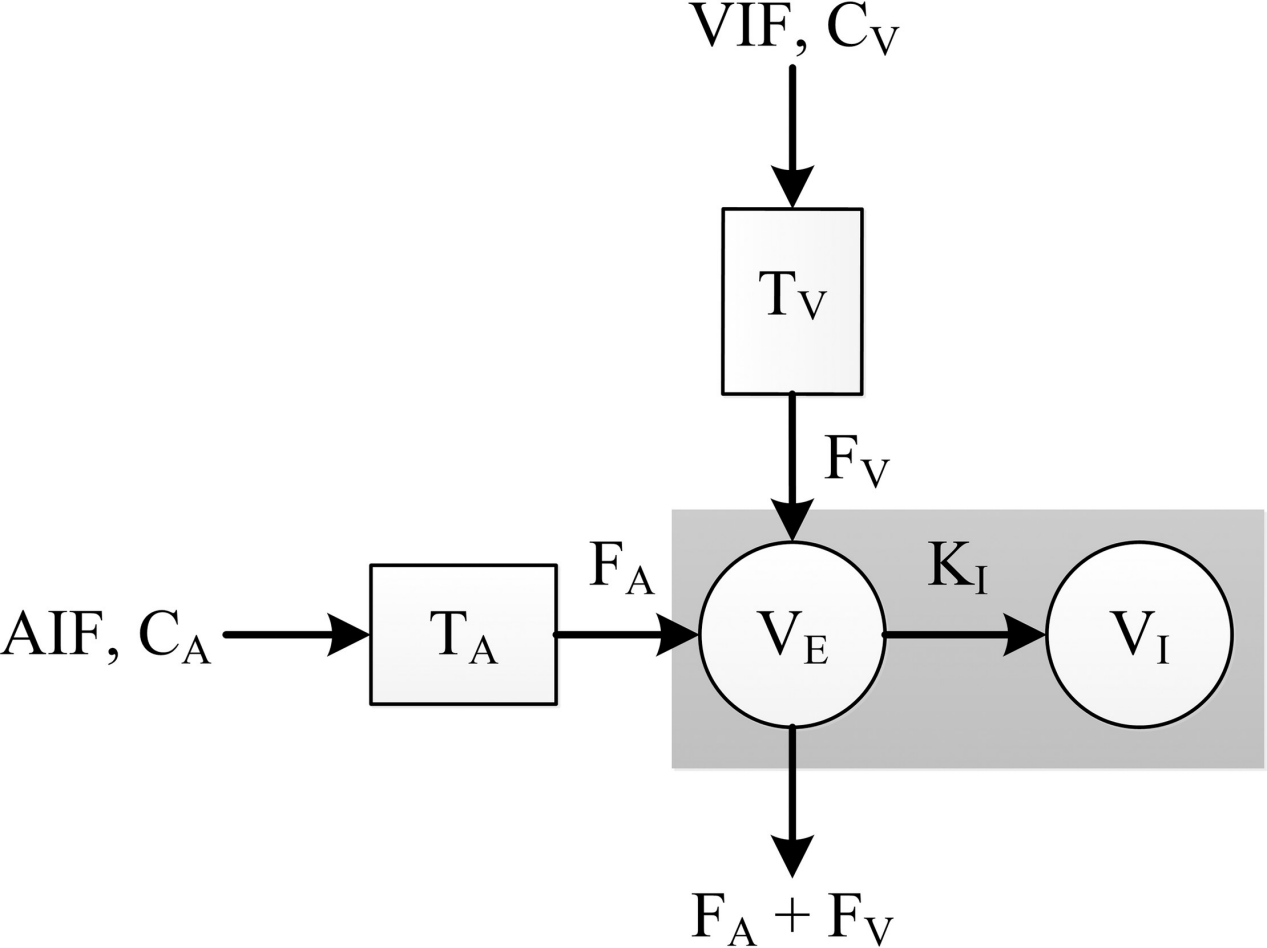
Description for Sourbron’s model. It is a dual-inlets, two-compartment uptake model for Gadoxetate disodium in the liver. The AIF (C_A_) and VIF (C_V_) are dual inlets into the liver, representing the concentration of the contrast agent over time entering from the hepatic artery and the portal vein. *T*_A_ and *T*_V_ are time delays. *F*_A_ and *F*_V_ are the arterial and venous plasma flows, respectively (in milliliters per minute per 100 mL). The gray rectangle represents the liver, the left circle denotes the extravascular extracellular compartment *V*_E_ (in milliliters per 100 mL) and the right circle stands for the hepatocytes, i.e. the extravascular intracellular compartment *V*_I_. *K*_I_ (per minute) is the liver uptake rate.

Sourbron *et al*. [2] developed a dual-inlet, two-compartment uptake model that was especially designed for the intracellular hepatobiliary contrast agent Primovist. The diagram in Fig 3 illustrates the model. The arterial input function *C*_A_ and venous input function *C*_V_ are the dual inlets representing the contrast agent concentration in the blood plasma supplied to the liver by the hepatic artery and the portal vein, respectively. *T*_A_ and *T*_V_ represent time delays and *F*_A_ and *F*_V_ are constants representing the volume transfer rates from the plasma compartments into the extravascular, extracellular space. Furthermore, the gray rectangle denotes liver tissue, the left circle represents the extravascular extracellular compartment and the right circle stands for the extravascular intracellular compartment, i.e. corresponding to the hepatocytes. As such, *V*_E_ is the extravascular extracellular volume and *K*_I_ represents the uptake rate of the hepatocytes represented by a volume *V*_I_.

The analytical solution of Sourbron’s model yielding the total contrast agent concentration *C*_T_ in a voxel is

$$\begin{array}{l} C_{T}\left(t\right)=\frac{K_{I}}{F_{A}+F_{V}+K_{I}}\int_{0}^{t}\left[F_{A}C_{A}\left(\tau -T_{A}\right)+F_{V}C_{V}\left(\tau -T_{V}\right)\right]d\tau \\ \;+\frac{F_{A}+F_{V}}{F_{A}+F_{V}+K_{I}}e^{-\frac{t}{T_{E}}}\int_{0}^{t}e^{\frac{\tau }{T_{E}}}\left[F_{A}C_{A}\left(\tau -T_{A}\right)+F_{V}C_{V}\left(\tau -T_{V}\right)\right]d\tau , \end{array}$$ (8)

where

$$T_{E}=\frac{V_{E}}{F_{A}+F_{V}+K_{I}}.$$

A derivation of this expression can be found in S1 Appendix.

### The combined Orton-Sourbron (COS) model

Since vascular input functions are the front-ends of Sourbron’s liver model, a comprehensive model can be derived by inserting Eq (7) into Eq (8). This leads for the contrast agent concentration in a voxel *C*_*T*,*I*_ due to either AIF or VIF (i.e *I*∈{*A*,*V*} to:

$$C_{T,I}\left(t\right)=F_{I}\left(\begin{array}{l} +A_{B}\frac{\mu_{B}V_{E}-K_{I}}{\mu_{B}\left(F_{A}+F_{V}+K_{I}-\mu_{B}V_{E}\right)}\left(t-T_{I}\right)e^{-\mu_{B}\left(t-T_{I}\right)}\\ -\left(\begin{array}{l} +A_{B}\frac{{\left(\mu_{B}V_{E}-K_{I}\right)}^{2}+\left(F_{A}+F_{V}\right)K_{I}}{\mu_{B}^{2}{\left(F_{A}+F_{V}+K_{I}-\mu_{B}V_{E}\right)}^{2}}\\ +A_{G}\frac{\mu_{B}V_{E}-K_{I}}{\mu_{B}\left(F_{A}+F_{V}+K_{I}-\mu_{B}V_{E}\right)} \end{array}\right)e^{-\mu_{B}\left(t-T_{I}\right)}\\ +A_{G}\frac{\mu_{G}V_{E}-K_{I}}{\mu_{G}\left(F_{A}+F_{V}+K_{I}-\mu_{G}V_{E}\right)}e^{-\mu_{G}\left(t-T_{I}\right)}\\ +\frac{\left(F_{A}+F_{V}\right)V_{E}^{2}}{F_{A}+F_{V}+K_{I}}\left(\begin{array}{l} +A_{B}\frac{1}{{\left(F_{A}+F_{V}+K_{I}-\mu_{B}V_{E}\right)}^{2}}\\ +A_{G}\frac{\left(\mu_{B}-\mu_{G}\right)}{\left(F_{A}+F_{V}+K_{I}-\mu_{B}V_{E}\right)\left(F_{A}+F_{V}+K_{I}-\mu_{G}V_{E}\right)} \end{array}\right)e^{-\frac{F_{A}+F_{V}+K_{I}}{V_{E}}\left(t-T_{I}\right)}\\ +\frac{K_{I}}{F_{A}+F_{V}+K_{I}}\left(\frac{A_{B}}{\mu_{B}^{2}}-\frac{A_{G}}{\mu_{B}}+\frac{A_{G}}{\mu_{G}}\right) \end{array}\right),$$ (9)

in which Orton’s model parameters (*μ*_*B*_,*μ*_*G*_) are particular for either AIF or VIF; *T*_I_ refers to the time delay associated with the particular input function. A derivation of this expression can be found in S2 Appendix.

The final model is expressed as the sum of contributions from AIF and VIF:

$$C_{T}\left(t\right)=C_{T,A}\left(t\right)+C_{T,V}\left(t\right),$$ (10)

in which *C*_*T*_, as before, models the total contrast agent concentration in a voxel.

Practically, we set the time delay of the portal vein (T_V_) to zero (as in [2]) since it is smaller than the temporal resolution of our data (2.2 s). We do estimate the time delay of the arterial input function (T_A_), which is larger as it is measured in the aorta, i.e. further away from the liver.

### Varying effective flip-angle compensation

Fig 2 shows the distribution of TICs for a particular patient. Several abrupt drops in signal intensity may be observed that appear correlated with the liver’s displacement.

We hypothesize that this signal variation can be modeled as a deviation in the locally applied flip-angle. In general, the signal intensity in a voxel emanating from a gradient echo sequence, neglecting $T_{2}^{*}$ decay, and assuming the spins are in the steady state, is given by:

$$S\left(\alpha ,T_{1}\right)=N\left(H\right)\sin\left(\alpha \right)\frac{1-e^{-\frac{TR}{T_{1}}}}{1-\cos\left(\alpha \right)e^{-\frac{TR}{T_{1}}}},$$ (11)

where *N*(*H*) is the local proton density multiplied by an arbitrary factor (the scaling factor used by the scanner), *T*_1_ the spin-lattice relaxation time, α the flip-angle and *TR* the repetition time.

Furthermore, the Relative Signal Intensity (RSI) in a voxel while the contrast agent is flowing in can be expressed as:

$$RSI\left(\alpha ,T_{1}\right)=\frac{S\left(\alpha ,T_{1}\right)}{S\left(\alpha_{0},T_{10}\right)}=\frac{sin\left(\alpha \right)\frac{1-e^{-\frac{TR}{T_{1}}}}{1-\cos\left(\alpha \right)e^{-\frac{TR}{T_{1}}}}}{sin\left(\alpha_{0}\right)\frac{1-e^{-\frac{TR}{T_{10}}}}{1-\cos\left(\alpha_{0}\right)e^{-\frac{TR}{T_{10}}}}},$$ (12)

in which α_0_ is the presumed flip-angle in the voxel prior to contrast administration) (we assume 15°, i.e. the flip angle as per scan protocol); *T*_10_ the spin-lattice relaxation time before contrast arrives, *T*_1_ the actual spin-lattice relaxation time and α the actually perceived flip-angle during the dynamic scan, modeling the effect of a deviating flip angle.

The contrast agent concentration *C*_T_ can be expressed as a function of α, *T*_1_ and the RSI as (see S3 Appendix):

$$C_{T}\left(\alpha ,T_{1}\right)=\frac{1}{R}\left[-\frac{1}{TR}\ln\left(\frac{\frac{1-\cos\left(\alpha_{0}\right)e^{-\frac{TR}{T_{10}}}}{1-e^{-\frac{TR}{T_{10}}}}-RSI\left(\alpha ,T_{1}\right)}{\frac{1-\cos\left(\alpha_{0}\right)e^{-\frac{TR}{T_{10}}}}{1-e^{-\frac{TR}{T_{10}}}}-RSI\left(\alpha ,T_{1}\right)\cos\left(\alpha_{0}\right)}\right)-\frac{1}{T_{10}}\right],$$ (13)

with *R* the relaxivity of the applied contrast agent (for Gd-EOB-DTPA at 3T, *R* = 7 s^-1^mM^-1^l [24]).

Consequently, the error in the calculated contrast agent concentration due to deviating flip-angle (e.g. caused by B1-inhomogeneity) is:

$$\Delta C_{T}\left(\alpha ,T_{1}\right)=C_{T}\left(\alpha ,T_{1}\right)-C_{T}\left(\alpha_{0},T_{1}\right).$$ (14)

**Fig 4:**
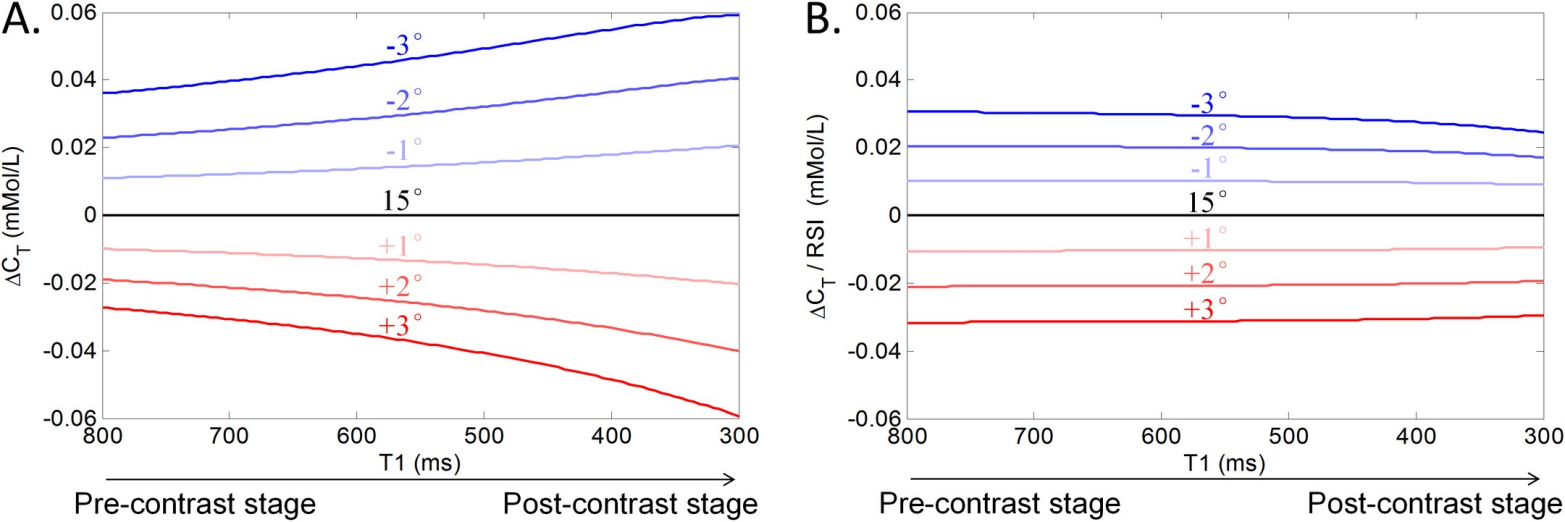
Varying flip-angle’s influence on contrast agent concentration and its correction. (A) Error in the contrast agent concentration due to a deviation in flip-angle (e.g. due to B1 inhomogeneity) as a function of T_1_ value; (B) Error in contrast agent concentration after normalization by the relative signal intensity.

The intrinsic T_1_ value of the liver prior to contrast injection is around 800 ms [25], while we estimate that the effective T_1_ can be as small as 300 ms after contrast injection. Fig 4(A) shows Δ*C*_*T*_ for this range of *T*_1_ values as well as for flip angle deviations varying from -3^o^ to +3^o^. Essentially, the graph demonstrates that the error in *C*_T_ is non-linearly dependent on *T*_1_ for any given deviation in flip-angle. However, normalizing through division by RSI(α, T_1_) yields profiles that are independent of *T*_1_ for every flip-angle deviation, see Fig 4(B). Furthermore, the distance between the profiles reflects that there is an approximately linear relation between Δ*C*_*T*_ and the applied flip-angle.

We model the contrast agent concentration in a voxel as:

$$C_{T}{}^{\prime }\left(t\right)=C_{T}\left(t\right)+\left[\alpha RSI(t)\;\beta RSI(t)\;\gamma RSI(t)\right]{\left[\Delta u\left(t\right)\;\Delta v\left(t\right)\;\Delta w\left(t\right)\right]}^{T},$$ (15)

in which *C*’_T_ (*t*) is the measured, uncorrected contrast agent concentration in a voxel; *C*_T_ (*t*) is the combined Orton-Sourbron (COS) model, see Eq (10); *α*, *β* and *γ* are proportionality constants that need to be estimated and *RSI(*t) is the relative signal intensity with respect to the one in the pre-contrast stage, i.e. *S*(α, *T*_1-post_)/*S*(α, *T*_1-pre_). [Δ*u*(*t*) Δ*v*(*t*) Δ*w*(*t*)] is the estimated displacement of the considered voxel in the dynamic at time *t*, relative to the last dynamic. This estimated displacement is taken from the deformation field emanating from the registration of the dynamic at time *t* to the last dynamic. As such a linear relation was fit between the displacement of liver and the modeled deviation contrast agent concentration as a first order approximation.

Thus, by fitting Eq (15) to the concentration curves we have not only parameterized the arrival time in Sourbron’s model (through the COS approach), but also included an implicit varying flip-angle correction (FLAC). Henceforth, we will refer to this as our COS-FLAC approach.

### Experimental setup

#### Assessment of registration performance

The correctness of each registration was first visually checked. Furthermore, synthetic MR images were generated by artificially deforming the last image of the DCE series, i.e. the fixed images of our registration procedure, and then registering the deformed images back to the originals. The artificial deformations were generated by randomly selecting *10* estimated deformations fields from the DCE series. As such, the ground truth is known (the originals), enabling to calculate the mean target registration error (mtre) for each point in the liver. We did so since it appeared not feasible to reliably identify landmarks in these data. This was due to the low resolution of the data and absence of highly characteristic points around the liver in our data.

#### Comparison between Sourbron’s model and the COS model

We first ran a numerical experiment to compare the accuracy and time efficiency of Sourbron’s original approach and the proposed COS technique.

Essentially, synthetic data was generated in two steps: a *parameter estimation* step and a *data generation* step.

In the *parameter estimation* step the input function parameters (of AIF and VIF) were first obtained by fitting Orton’s model in a small region of interest in the aorta respectively the portal vein in each patient. Subsequently, the PKM parameters were estimated for both the reference approach (Sourbron’s) and the proposed method in each liver voxel. Then, the PKM parameters of the two methods were averaged (to be unbiased) and this average was taken as the ground truth.

As such, known input function parameters were obtained from each patient as well as known PKM parameters from each liver voxel.

Subsequently, in the *data generation step* synthetic data was generated by (1) creating ground truth input functions from the estimated Orton’s model parameters (of AIF and VIF) and adding noise; (2) generating tissue TCC’s from the ground truth PKM parameters and adding noise. The standard deviation of the added noise on the input functions equaled the root mean square error (RMSE) of Orton’s model fit; it was set to the average RMSE of the reference and proposed model fits for the TCC’s.

Thus, a wide variety of artificial, noisy time intensity curves could be generated (for each liver voxel one such curve). Please note that the synthetic data was generated by averaging the PKM parameters of reference and proposed method exactly to avoid a bias to either approach.

Finally, we fitted both PK models to the noisy synthetic data and compared the estimated PK model parameters with the ground truth. The nonlinear least-squares fitting routine *lsqcurvefit* in MATLAB (version R2015b; Mathworks, Natick, USA) was used to perform the model fits; 19 cores were adopted for parallel computing on a HPC equipped with two Intel(R) Xeon(R) CPU E5-2698 v4 clocked at 2.20GHz and 256GB RAM memory.

#### Relation between displacement and programmed flip-angle deviations

Eq (15) assumed that a difference from the true contrast agent is linearly related to the displacement of a liver voxel. Furthermore, the difference (Δ*C*_*T*_) was modeled to linearly relate to the deviation from the programmed flip-angle (Fig 4).

To assess the validity of this, the zeta-map from the DREAM sequence, representing the deviation from the programmed flip angle, was geometrically aligned to the first dynamic. Observe that the displacement of a liver voxel in any DCE image is given by the registration transformation that is relative to the first dynamic. Subsequently, the difference in zeta value over the displacement vector (Δ*zeta*) was correlated to the displacement across all dynamics. The strength of the correlation was assessed by Spearman correlation coefficient and the significance of the correlation was determined.

#### The COS-FLAC model with and without RSI weighting

Models of increasing complexity, from the COS-model up to the COS-FLAC model with RSI weighting, were fit to the data of the 11 subjects described in Section *Varying effective flip-angle compensation*. The root mean square error (RMSE) of the residual that remains after fitting the COS and the COS-FLAC models to the signal were determined in order to quantitatively assess the performance. However, increasing degrees of freedom by adding parameters to a model generally leads to decreased smaller RMSE of the fit residual. To evaluate whether the added parameters truly contributed to a better fit, three model-selection criteria were applied: Akaike’s information criterion (AIC) [26], the Bayesian information criterion (BIC) [27], and Information Complexity (ICOMP) [28].

## Results

### Assessment of registration performance

**Fig 5:**
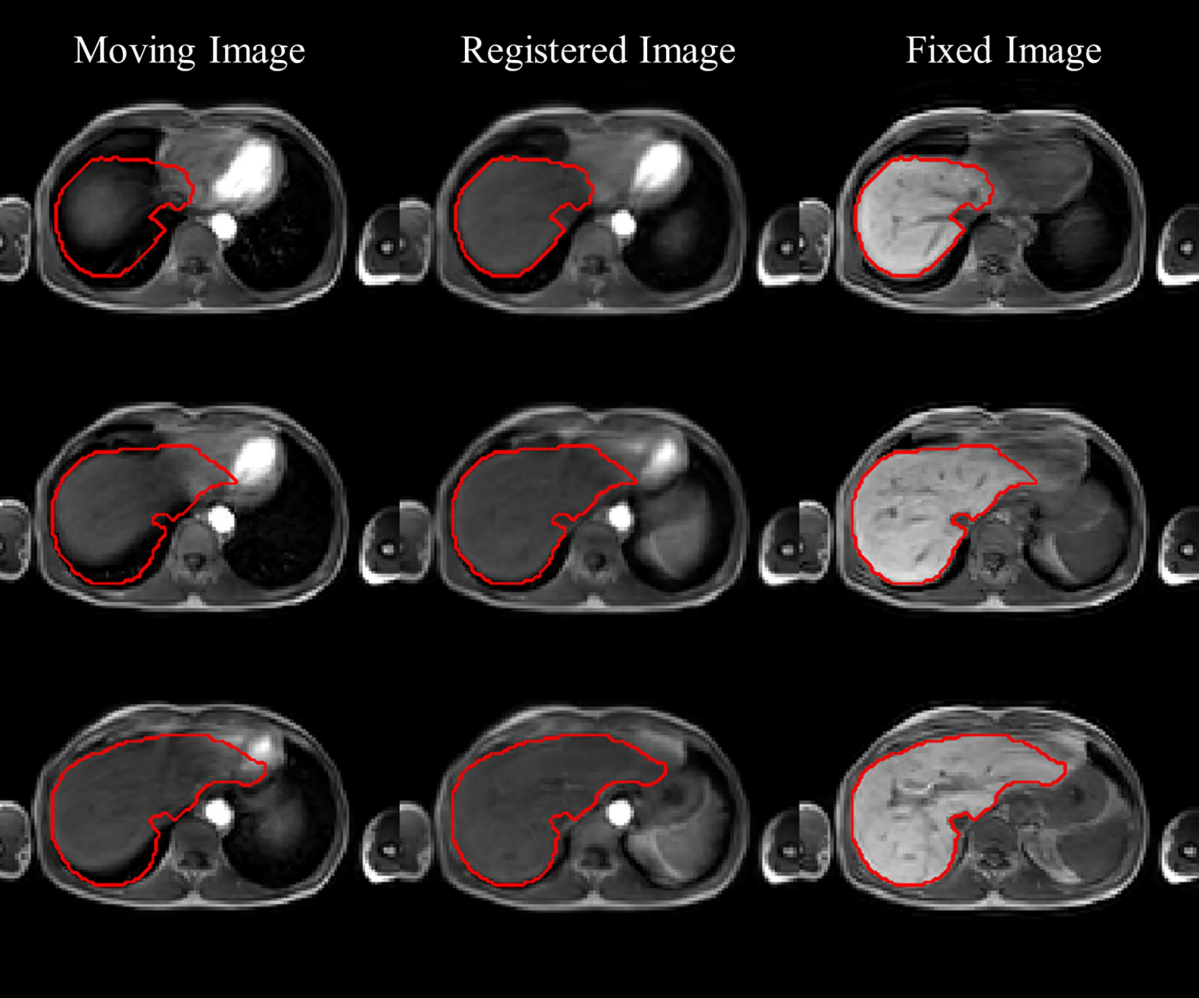
Illustration of registration performance. Moving image (first column), registered images obtained with our registration method (second column) and the fixed image (last column). Three (non-consecutive) slices were chosen (from top to bottom). In each image the outline of the liver from the fixed image is superimposed.

A typical example of illustrating the performance of the registration algorithm is contained in Fig 5.

Furthermore, we found that the mtre across the selected deformation fields and patients was 1.3269 mm with a standard deviation of 0.6905 mm. The unregistered data yielded an mtre of 8.0234 mm with a standard deviation of 7.4431 mm. As such, these quantitative results confirm the accurate performance of the image registration based on the visual assessment.

### Fitting results of input function models

Orton’s model is a general model to describe an organ’s AIF. For reference, the fitting parameters of Orton’s model as well as two measures of the goodness of the fit for both AIF and VIF in each patient is contained in the S4 Appendix.

### Comparison between Sourbron’s model and the COS model

Table 2 shows the mean difference between the ground truth and estimated PK model parameters (as well as corresponding standard deviations) for Sourbron’s model and the COS model. It shows that the COS model achieved smaller mean difference *and* standard deviation on four PK model parameters out of five. Additionally, the COS model was fitted more than 7 times faster than Sourbron’s model due to the analytical representation of AIF and VIF.

**Table 2:** Comparison between Sourbron’s model (discrete AIF) and COS model (analytical AIF) in terms of estimating PK model parameters and time efficiency on synthetic data. The numbers report the mean difference from the ground truth and corresponding standard deviation (between brackets). The numbers printed in boldface are the best outcomes per row.

	Original Sourbron's model	COS model
Δ*F*_A_ (ml / min / 100ml)	2.058 (3.983)	**-0.096 (2.297)**
Δ*F*_V_ (ml / min / 100ml)	-17.318 (29.495)	**6.965 (23.260)**
Δ*K*_I_ (/ 100 / min)	0.407 (1.407)	**0.339 (0.704)**
Δ*V*_E_ (ml / 100 ml)	**0.005 (0.047)**	-0.011 (0.038)
Δ*T*_A_ (sec)	0.172 (1.409)	**0.073 (1.330)**
Computation time (min)	13.257 (6.018)	**1.783 (0.492)**

### Relation between displacement and programmed flip-angle deviations

Table 3 collates the mean correlation coefficients averaged over all liver voxels for each patient. Additionally, the mean p-values (and associated standard deviations) of the correlations are given. The p-values are corrected via the Benjamini–Hochberg procedure [29] for multiple testing, The false discovery rate used for Benjamini-Hochberg correction in our paper is 0.05. The mean adjusted p-values demonstrate that the correlations are highly significant. Furthermore, the correlation coefficients indicated a moderate to strong linear relationship [30]. The moderate to strong correlation and the significance of the correlations are indications that the assumption is appropriate.

**Table 3:** Mean Spearman correlation coefficients (and associated standard deviation) of the correlations between the displacement and the deviation from the applied flip-angle over all liver voxels as well as the mean p-values (and standard deviation) of these correlations stratified by patient number.

Case	Correlation coefficients	Adjusted P-values	The number of voxels
**1**	0.724 (0.253)	0.0010 (0.0024)	11439
**2**	0.555 (0.254)	0.0011 (0.0025)	11249
**3**	0.613 (0.262)	0.0010 (0.0024)	35818
**4**	0.485 (0.251)	0.0038 (0.0081)	28783
**5**	0.556 (0.275)	0.0037 (0.0080)	13581
**6**	0.639 (0.120)	0.0000 (0.0000)	13333
**7**	0.692 (0.262)	0.0003 (0.0006)	16979
**8**	0.726 (0.199)	0.0004 (0.0009)	14508
**9**	0.498 (0.276)	0.0083 (0.0171)	26092
**10**	0.590 (0.240)	0.0003 (0.0007)	21681
**11**	0.758 (0.123)	0.0002 (0.0005)	13881
**Overall**	0.595 (0.262)	0.0013 (0.0031)	18849 (8155)

### The COS-FLAC model with and without RSI weighting

**Fig 6:**
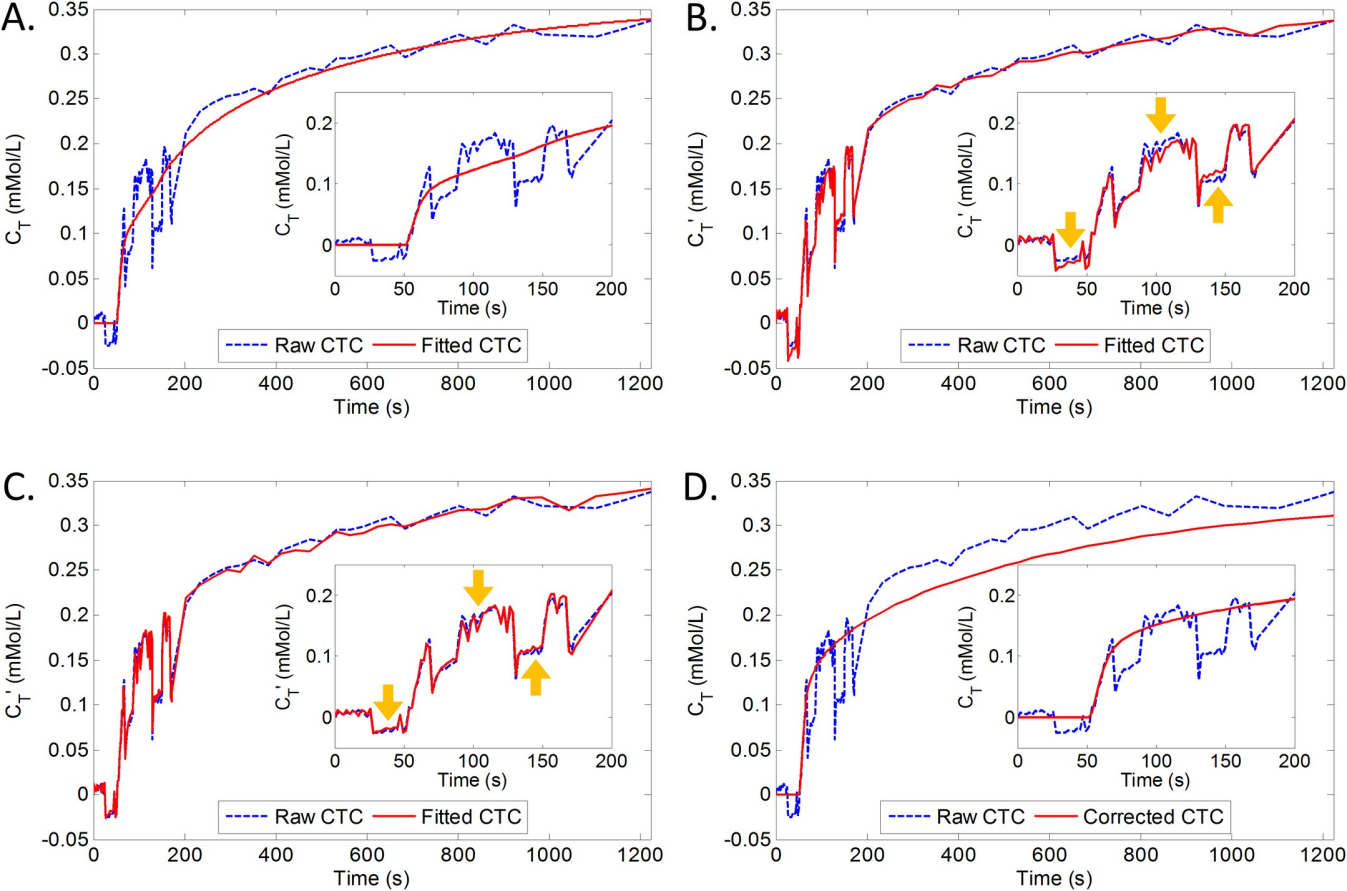
The fitting results of different models. Fitting results (red) of different models to the concentration-time curve (*C*’_T_ (*t*), blue) extracted from a single voxel. (A) Combined Orton-Sourbron (COS) model; (B) COS model with varying flip-angle correction (FLAC) but without the RSI weighting term in Eq (15); (C) COS-FLAC model including the RSI weighting term; (D) Pure concentration-time curve (*C*_T_ (*t*)) recovered from (C). All sub-plots show zoom-ins of the initial part of the curves, i.e. 0–200 s.

The signal intensity in the liver of one patient was already shown in Fig 2(C). Fig 6 illustrates how models (red) of increasing complexity, from COS up to the COS-FLAC model with RSI weighting, fit to the concentration curve (blue) from an exemplary voxel. Insets show zoom-ins of the initial part of the graphs, containing most of the breath holds. In Fig 6(A) merely the combined Orton-Sourbron (COS) model was fitted. The model does not fit the strong fluctuations of the first part of the concentration curve. In Fig 6(B), we fitted the COS model with varying flip-angle correction (FLAC) model but without the RSI weighting term. Clearly, an improved fitting result was achieved compared with Fig 6(A). However, some parts of the concentration curve are slightly off, see the yellow arrows in Fig 6(B). Fig 6(C) shows that the full COS-FLAC model including the RSI weighting term achieved an even better fit. For reference, Fig 6(D) shows the mere concentration part *C*_*T*_ from Eq (14) taken from the fit in Fig 6(C).

The mean RMSEs of fitting in all 11 patients is collated in Table 4. It shows that the COS-FLAC model with RSI weighting term achieved the lowest RMSE, which is significantly better than the COS model and the COS-FLAC model without RSI weighting term (p <0.001, assessed by paired t-tests, and corrected via the Benjamini–Hochberg procedure [29] for multiple testing). Henceforth the, COS-FLAC model refers to the model including the RSI weighting term.

**Table 4:** Average root mean square error (RMSE) of the residual that remains after fitting the COS and COS-FLAC models with and without RSI weighting term. The numbers are the mean value and the standard deviation (std) averaged over all liver voxels. The best results are printed in boldface.

Case	COS	COS-FLAC without RSI weighting	COS-FLAC with RSI weighting
**1**	1.902E-02 (4.334E-03)	1.496E-02 (3.652E-03)	**1.461E-02 (3.678E-03)**
**2**	1.600E-02 (5.355E-03)	1.160E-02 (3.100E-03)	**1.129E-02 (3.089E-03)**
**3**	2.664E-02 (1.117E-02)	2.054E-02 (7.739E-03)	**2.012E-02 (7.579E-03)**
**4**	4.260E-02 (1.740E-02)	3.523E-02 (1.441E-02)	**3.491E-02 (1.441E-02)**
**5**	2.206E-02 (5.618E-03)	1.952E-02 (4.966E-03)	**1.929E-02 (4.927E-03)**
**6**	2.975E-02 (1.176E-02)	2.059E-02 (6.565E-03)	**1.948E-02 (5.886E-03)**
**7**	2.192E-02 (9.294E-03)	1.111E-02 (3.280E-03)	**1.067E-02 (3.325E-03)**
**8**	2.500E-02 (9.045E-03)	1.890E-02 (6.708E-03)	**1.826E-02 (6.708E-03)**
**9**	1.688E-02 (4.040E-03)	1.411E-02 (3.953E-03)	**1.406E-02 (3.984E-03)**
**10**	2.338E-02 (5.071E-03)	2.033E-02 (4.746E-03)	**2.005E-02 (4.728E-03)**
**11**	3.243E-02 (9.060E-03)	1.916E-02 (5.164E-03)	**1.802E-02 (5.152E-03)**
**Overall**	2.515E-02 (1.047E-02)	1.868E-02 (7.446E-03)	**1.819E-02 (7.319E-03)**

Table 5 shows the scores that PK models get according to three model selection criteria as well as the percentage of voxels in which these criteria favored the COS-FLAC model over the mere COS approach. The proposed COS-FLAC technique was considered to yield a better fit in the majority of voxels across all subjects according to all three model-selection methods (*p* <0.001, assessed by paired t-tests).

**Table 5:** Mean scores in the liver of 11 subjects that models got according to three model-selection criteria as well as the percentage of the in which the COS-FLAC model outperformed the COS approach: Akaike’s Information criterion (AIC), Bayesian Information Criterion (BIC) and Information Complexity (ICOMP). A lower score indicates the model fits the data better. The COS and COS-FLAC models involve 5 and 8 parameters, respectively, as is also indicated in the table.

Case	AIC			BIC			ICOMP		
	COS (5)	COS-FLAC (8)	COS-FLAC < COS (%)	COS (5)	COS-FLAC (8)	COS-FLAC < COS (%)	COS (5)	COS-FLAC (8)	COS-FLAC < COS (%)
**1**	-852.679 (51.460)	**-908.107 (57.626)**	98.615	-839.268 (51.460)	**-886.650 (57.626)**	93.292	-853.657 (51.386)	**-911.632 (57.138)**	99.716
**2**	-893.338 (78.885)	**-963.347 (62.107)**	97.817	-879.927 (78.885)	**-941.890 (62.107)**	90.685	-894.220 (79.409)	**-968.002 (62.020)**	99.388
**3**	-776.654 (103.998)	**-833.659 (92.809)**	98.159	-763.243 (103.998)	**-812.201 (92.809)**	91.089	-780.678 (104.698)	**-841.156 (93.754)**	99.499
**4**	-673.668 (94.404)	**-712.605 (94.814)**	95.422	-660.351 (94.404)	**-691.297 (94.814)**	82.586	-674.465 (94.602)	**-717.599 (93.913)**	98.769
**5**	-820.607 (58.029)	**-848.015 (57.665)**	94.591	-807.197 (58.029)	**-826.558 (57.665)**	79.425	-820.605 (58.159)	**-851.622 (57.673)**	98.952
**6**	-767.001 (91.726)	**-848.749 (68.966)**	98.667	-753.591 (91.726)	**-827.292 (68.966)**	94.646	-767.590 (92.134)	**-853.252 (68.769)**	99.558
**7**	-833.484 (98.759)	**-975.161 (71.903)**	99.371	-820.073 (98.759)	**-953.704 (71.903)**	97.271	-833.528 (98.651)	**-977.555 (71.784)**	99.695
**8**	-795.408 (83.835)	**-860.269 (84.557)**	97.925	-781.997 (83.835)	**-838.812 (84.557)**	91.634	-794.805 (83.843)	**-861.989 (84.349)**	99.047
**9**	-878.362 (54.731)	**-917.277 (64.419)**	97.159	-864.951 (54.731)	**-895.820 (64.419)**	88.554	-879.493 (55.079)	**-922.729 (64.564)**	99.458
**10**	-808.133 (48.258)	**-840.045 (53.112)**	96.209	-794.722 (48.258)	**-818.588 (53.112)**	84.253	-809.190 (48.543)	**-844.428 (53.273)**	98.985
**11**	-738.370 (67.289)	**-863.216 (66.717)**	99.083	-724.959 (67.289)	**-841.759 (66.717)**	96.129	-736.598 (67.654)	**-868.164 (66.278)**	99.616
**Overall**	-794.126 (101.212)	**-860.240 (100.905)**	97.397	-780.728 (101.198)	**-838.802 (100.882)**	89.380	-794.990 (101.466)	**-864.651 (100.638)**	99.300

Previously, Sourbron *et al* [2], Chandarana *et al* [31] and Simeth *et al* [32] reported on liver uptake rates based on their respective PK models, see Table 6. Our results are a little bit higher than those in previous studies but are still in the same order.

**Table 6:** Comparison with literature values regarding liver uptake rate.

data source	Liver uptake rate in normal-appearing liver tissue (/ min)	Liver uptake rate in lesions (/ min)
Sourbron *et al*	3.4 (1.9)	1.7 (1.4)
Chandarana *et al*	6.53 (2.4)	3.03 (2.1)
Simeth *et al*	7.44 (4.93)	-
Our paper	8.23 (5.43)	6.47 (15.83)

## Discussion

In this paper, we proposed an improved pharmacokinetic model for DCE-MRI of the liver. The novelties of our work comprise: (1) analytically modeling the arrival-time of the contrast agent in a voxel; (2) compensation for effects that can be modeled by allowing for a breath-dependent B1-induced variation of the experienced flip-angle in each voxel.

The VIF and AIF might not be completely independent functions, which could introduce correlations in the parameter estimation. Clearly, they were measured in different arteries and we have observed different shapes in our data. For this reason, we modelled them independently.

Orton’s model was adopted to represent the liver’s input functions (hepatic artery and portal vein) and embed them into Sourbron’s model. The combined Orton and Sourborn (COS) model was shown to enhance the fitting accuracy as well as the efficiency of the model fitting (see Table 2). The poorer performance of Sourbron’s original approach is due to the discretized delay of the arterial input and determining the best model fit over a set of delay values.

A potentially deviating flip-angle was modeled to linearly relate to the displacement of a liver voxel with respect to the first image. We referred to the approach combining both novelties as the COS-FLAC model. The validity of our approach is supported by the moderate to strong linear correlation between displacement and deviation in flip angle. There are some weak correlations in part of the voxels in all cases. We observed that the voxels showing the weak correlation generally do not exhibit large displacements across the time series. In other words, these voxels do not move much. In these cases, the corresponding deviation in flip angles typically was also not large. As a result, the correlation between them is low. Observe that the low correlations in these voxels are not incompatible with our approach: a small displacement in these voxels will produce only a very small signal correction.

One may observe that the same, noisy AIF and VIF were at the basis of estimating the PKM parameters with the two methods. However, a crucial difference is in how the methods deal with arrival time. The errors in the arrival times indeed are small: see Table 2. Simultaneously, larger errors in the other parameters can be observed for the reference method. We attribute this to the correlation with the arrival time. Indeed, with the arrival time constrained to the ground truth value, much smaller errors in the other parameters were observed (data not shown).

The COS-FLAC model was quantitatively assessed by the root mean square error (RMSE) of the residual that remains after fitting the model to the signal in every voxel of the liver. We found that the COS-FLAC model achieved significantly lower RMSE than the COS approach. Furthermore, three model complexity criteria showed that the COS-FLAC model outperformed the COS model in the vast majority of voxels. These findings confirm that a small degree of B1-inhomogeneity can have a marked effect on the estimation of PKM parameters, cf. [14][15].

One might argue that the COS approach would suffice in voxels in which there is no deviation in flip-angle. This might explain why, according to the model selection criteria, there are still some voxels in which this simpler model appears sufficient. At the same time, the large number of voxels in which the COS-FLAC approach is favored, emphasizes to our opinion its importance.

There are several limitations of our work. A first limitation is that the number of subjects is rather small. Clearly, evaluating the performance of the method on a larger number of subjects would be more convincing. Unfortunately, we are restricted to a small number of subjects as our work is part of a pilot study into the uptake rate of the contrast medium into liver cells.

A second limitation is the lack of a reference standard. Obtaining the true pharmacokinetic tissue parameters under realistic measurement circumstances is a highly complex, still unsolved issue.

## Supporting information

S1 Appendix Derivation of Sourbron’s model. (DOCX)

S2 Appendix Derivation of COS model. (DOCX)

S3 Appendix Derivation from signal intensity to tissue concentration. (DOCX)

S4 Appendix Fitting results of input function models. (DOCX)
