# Supplementary material for: A pharmacokinetic model including arrival time for two inputs and compensating for varying applied flip-angle in dynamic gadoxetic acid-enhanced MR imaging
Source: PLoS One. 2019 Aug 15;14(8):e0220835. doi: 10.1371/journal.pone.0220835 (PMC6695151; doi:10.1371/journal.pone.0220835)
Supplement: S1 Appendix — (DOCX) [file pone.0220835.s001.docx]

**S1 Appendix. Derivation of Sourbron’s model**

Sourbron’s model:

$$\left\{\begin{matrix} V_{E}\frac{dC_{E}\left(t\right)}{dt}=F_{A}C_{A}\left(t-T_{A}\right)+F_{V}C_{V}\left(t-T_{V}\right)-\left(F_{A}+F_{V}+K_{I}\right)C_{E}\left(t\right) \\ V_{I}\frac{dC_{I}\left(t\right)}{dt}=K_{I}C_{E}\left(t\right) \\ C_{T}\left(t\right)=V_{E}C_{E}\left(t\right)+V_{I}C_{I}\left(t\right) \end{matrix}\right.,$$

where

F_A_: Arterial plasma flow (mL/min/100 mL)

F_V_: Venous plasma flow (mL/min/100 mL)

T_A_: Arterial delay (sec)

T_V_: Venous delay (sec)

K_I_: Uptake rate (per min)

V_E_: Extracellular volume (mL/100 mL)

V_I_: Hepatocyte volume (mL/100 mL)

T_E_: Extracellular mean transit time (sec)

The first equation in Sourbron’s model is given by

$$V_{E}\frac{dC_{E}\left(t\right)}{dt}=F_{A}C_{A}\left(t-T_{A}\right)+F_{V}C_{V}\left(t-T_{V}\right)-\left(F_{A}+F_{V}+K_{I}\right)C_{E}\left(t\right).$$

According to

$$T_{E}=\frac{V_{E}}{F_{A}+F_{V}+K_{I}},$$

Eq can be rearranged as

$$\frac{dV_{E}C_{E}\left(t\right)}{dt}=F_{A}C_{A}\left(t-T_{A}\right)+F_{V}C_{V}\left(t-T_{V}\right)-\frac{V_{E}C_{E}\left(t\right)}{T_{E}}.$$

The equation is of the type

$$\frac{dy\left(t\right)}{dt}+k\left(t\right)y\left(t\right)=Q\left(t\right),$$

whose general solution is

$$\left\{\begin{matrix} y\left(t\right)=y\left(a\right)e^{-A\left(t\right)}+e^{-A\left(t\right)}\int_{0}^{t} Q\left(u\right)e^{-A\left(u\right)}du \\ A\left(t\right)=\int_{0}^{t} k\left(u\right)du \end{matrix}\right.,$$

where *a* is arbitrary. In our case, *a* = 0, *y*(*a*) = *y*(0) = 0.

Substituting of

$$\left\{\begin{matrix} y\left(t\right)=V_{E}C_{E}\left(t\right) \\ k\left(t\right)=\frac{1}{T_{E}} \\ Q\left(t\right)=F_{A}C_{A}\left(t-T_{A}\right)+F_{V}C_{V}\left(t-T_{V}\right) \end{matrix}\right.,$$

into Eq (6) results in

$$C_{E}\left(t\right)=\frac{1}{V_{E}}e^{-\frac{t}{T_{E}}}\int_{0}^{t} Q\left(u\right)e^{\frac{u}{T_{E}}}du.$$

Now consider the third equation in Eq

$$C_{T}\left(t\right)=V_{E}C_{E}\left(t\right)+V_{I}C_{I}\left(t\right).$$

Obtaining the derivatives of both sides

$$\frac{dC_{T}\left(t\right)}{dt}=V_{E}\frac{dC_{E}\left(t\right)}{dt}+V_{I}\frac{dC_{I}\left(t\right)}{dt}.$$

Substituting the first and second equations of Eq

$$\begin{matrix} \frac{dC_{T}\left(t\right)}{dt}=F_{A}C_{A}\left(t-T_{A}\right)+F_{V}C_{V}\left(t-T_{V}\right)-\left(F_{A}+F_{V}+K_{I}\right)C_{E}\left(t\right)+K_{I}C_{E}\left(t\right) \\ =Q\left(t\right)-\left(F_{A}+F_{V}\right)C_{E}\left(t\right). \end{matrix}$$

The solution is

$$C_{T}\left(t\right)=\int_{0}^{t} \left[Q\left(t\right)-\left(F_{A}+F_{V}\right)C_{E}\left(t\right)\right]dt.$$

Substituting Eq (8) into Eq

$$\begin{matrix} C_{T}\left(t\right)=\int_{0}^{t} \left[Q\left(t\right)-\frac{F_{A}+F_{V}}{V_{E}}e^{-\frac{t}{T_{E}}}\int_{0}^{t} Q\left(u\right)e^{\frac{u}{T_{E}}}du\right]dt \\ =\int_{0}^{t} \left[Q\left(t\right)-\frac{F_{A}+F_{V}}{F_{A}+F_{V}+K_{I}}\frac{1}{T_{E}}e^{-\frac{t}{T_{E}}}\int_{0}^{t} Q\left(u\right)e^{\frac{u}{T_{E}}}du\right]dt \\ =\int_{0}^{t} Q\left(t\right)dt-\frac{F_{A}+F_{V}}{F_{A}+F_{V}+K_{I}}\frac{1}{T_{E}}\int_{0}^{t} e^{-\frac{t}{T_{E}}}\left(\int_{0}^{t} Q\left(u\right)e^{\frac{u}{T_{E}}}du\right)dt \\ =\int_{0}^{t} Q\left(t\right)dt+\frac{F_{A}+F_{V}}{F_{A}+F_{V}+K_{I}}\int_{0}^{t} \left(\int_{0}^{t} Q\left(u\right)e^{\frac{u}{T_{E}}}du\right)de^{-\frac{t}{T_{E}}} \\ =\int_{0}^{t} Q\left(t\right)dt+\frac{F_{A}+F_{V}}{F_{A}+F_{V}+K_{I}}\left\{\begin{matrix} \left.\left[\left(\int_{0}^{t} Q\left(u\right)e^{\frac{u}{T_{E}}}du\right)e^{-\frac{t}{T_{E}}}\right]\right|_{0}^{t} \\ -\int_{0}^{t} e^{-\frac{t}{T_{E}}}d\left(\int_{0}^{t} Q\left(u\right)e^{\frac{u}{T_{E}}}du\right) \end{matrix}\right\} \\ =\int_{0}^{t} Q\left(t\right)dt+\frac{F_{A}+F_{V}}{F_{A}+F_{V}+K_{I}}\left[\begin{matrix} \left(\int_{0}^{t} Q\left(u\right)e^{\frac{u}{T_{E}}}du\right)e^{-\frac{t}{T_{E}}} \\ -\int_{0}^{t} e^{-\frac{t}{T_{E}}}Q\left(t\right)e^{\frac{t}{T_{E}}}dt \end{matrix}\right] \\ =\int_{0}^{t} Q\left(t\right)dt+\frac{F_{A}+F_{V}}{F_{A}+F_{V}+K_{I}}\left(e^{-\frac{t}{T_{E}}}\int_{0}^{t} Q\left(u\right)e^{\frac{u}{T_{E}}}du-\int_{0}^{t} Q\left(t\right)dt\right) \\ =\frac{K_{I}}{F_{A}+F_{V}+K_{I}}\int_{0}^{t} Q\left(t\right)dt+\frac{F_{A}+F_{V}}{F_{A}+F_{V}+K_{I}}e^{-\frac{t}{T_{E}}}\int_{0}^{t} Q\left(u\right)e^{\frac{u}{T_{E}}}du, \end{matrix}$$

where

$$Q\left(t\right)=F_{A}C_{A}\left(t-T_{A}\right)+F_{V}C_{V}\left(t-T_{V}\right).$$
