# Supplementary material for: A pharmacokinetic model including arrival time for two inputs and compensating for varying applied flip-angle in dynamic gadoxetic acid-enhanced MR imaging
Source: PLoS One. 2019 Aug 15;14(8):e0220835. doi: 10.1371/journal.pone.0220835 (PMC6695151; doi:10.1371/journal.pone.0220835)
Supplement: S2 Appendix — (DOCX) [file pone.0220835.s002.docx]

**S2 Appendix. Derivation of COS model**

The AIF is described as:

$$\begin{matrix} C_{Orton}\left(t\right)=C_{B}\left(t\right)+C_{B}\left(t\right)*G\left(t\right) \\ =\left[A_{B}te^{-\mu_{B}t}+A_{G}\left(e^{-\mu_{G}t}-e^{-\mu_{B}t}\right)\right]u\left(t\right), \end{matrix}$$

where

$$\left\{\begin{matrix} A_{B}=a_{B}\mu_{B}^{2}\left(1-\frac{a_{G}}{\mu_{B}-\mu_{G}}\right) \\ A_{G}=\frac{a_{B}a_{G}\mu_{B}^{2}}{\left(\mu_{B}-\mu_{G}\right)^{2}} \end{matrix}\right..$$

The solution of Sourbron’s model is

$$\begin{matrix} C_{T}=\frac{K_{I}}{F_{A}+F_{V}+K_{I}}\int_{0}^{t} \left[F_{A}C_{A}\left(\tau-T_{A}\right)+F_{V}C_{V}\left(\tau-T_{V}\right)\right]d\tau\\ +\frac{F_{A}+F_{V}}{F_{A}+F_{V}+K_{I}}e^{-\frac{t}{T_{E}}}\int_{0}^{t} e^{\frac{\tau}{T_{E}}}\left[F_{A}C_{A}\left(\tau-T_{A}\right)+F_{V}C_{V}\left(\tau-T_{V}\right)\right]d\tau. \end{matrix}$$

Mainly there are two kinds of integrals inside

$$\left\{\begin{matrix} A\left(t\right)=\int_{0}^{t} F_{I}C_{I}\left(\tau-T_{I}\right)d\tau\\ B\left(t\right)=\int_{0}^{t} e^{\frac{\tau}{T_{E}}}F_{I}C_{I}\left(\tau-T_{I}\right)d\tau\end{matrix}\right.,$$

where I denotes either AIF (*C*_A_) and VIF (*C*_V_).

Now derive *A*(t) and *B*(t):

Substituting Eq and Eq into Eq we can obtain

$\begin{matrix} C_{T,I}=\frac{K_{I}}{F_{A}+F_{V}+K_{I}}\int_{0}^{t} \left[F_{A}C_{A}\left(\tau-T_{A}\right)+F_{V}C_{V}\left(\tau-T_{V}\right)\right]d\tau\\ +\frac{F_{A}+F_{V}}{F_{A}+F_{V}+K_{I}}e^{-\frac{t}{T_{E}}}\int_{0}^{t} e^{\frac{\tau}{T_{E}}}\left[F_{A}C_{A}\left(\tau-T_{A}\right)+F_{V}C_{V}\left(\tau-T_{V}\right)\right]d\tau\\ =\frac{K_{I}}{F_{A}+F_{V}+K_{I}}\left.\left\{F_{I}\left[\begin{matrix} -\frac{A_{B} }{\mu_{B}}\left(t-T_{0}\right) e^{-\mu_{B} \left(t-T_{I}\right)}+\left(\frac{A_{G} }{\mu_{B}}-\frac{A_{B} }{\mu_{B}^{2}}\right)e^{-\mu_{B} \left(t-T_{I}\right)} \\ -\frac{A_{G} }{\mu_{G}}e^{-\mu_{G} \left(t-T_{I}\right)}+\frac{A_{B}}{\mu_{B}^{2}}-\frac{A_{G}}{\mu_{B}}+\frac{A_{G}}{\mu_{G}} \end{matrix}\right]\right\}\right|_{I=A,V} \end{matrix}$ (8a)

$+\frac{F_{A}+F_{V}}{F_{A}+F_{V}+K_{I}}e^{-\frac{t}{T_{E}}}\left.\left\{F_{I}\left[\begin{matrix} +\frac{A_{B}T_{E}}{1-\mu_{B}T_{E}}\left(t-T_{0}\right)e^{\frac{t}{T_{E}}-\mu_{B} \left(t-T_{I}\right)} \\ -\frac{T_{E}}{1-\mu_{B}T_{E}}\left(\frac{A_{B}T_{E}}{1-\mu_{B}T_{E}}+A_{G} \right)e^{\frac{t}{T_{E}}-\mu_{B} \left(t-T_{I}\right)} \\ +\frac{A_{G}T_{E} }{1-\mu_{G}T_{E}}e^{\frac{t}{T_{E}}-\mu_{G} \left(t-T_{I}\right)} \\ +T_{E}\left(\frac{A_{G}}{1-\mu_{B}T_{E}}-\frac{A_{G}}{1-\mu_{G}T_{E}}+\frac{A_{B}T_{E}}{\left(1-\mu_{B}T_{E}\right)^{2}}\right)e^{\frac{T_{I}}{T_{E}}} \end{matrix}\right]\right\}\right|_{I=A,V}$ (8b)

$\begin{matrix} =\frac{K_{I}}{F_{A}+F_{V}+K_{I}}\left.\left\{F_{I}\left[\begin{matrix} -\frac{A_{B} }{\mu_{B}}\left(t-T_{0}\right) e^{-\mu_{B} \left(t-T_{I}\right)}+\left(\frac{A_{G} }{\mu_{B}}-\frac{A_{B} }{\mu_{B}^{2}}\right)e^{-\mu_{B} \left(t-T_{I}\right)} \\ -\frac{A_{G} }{\mu_{G}}e^{-\mu_{G} \left(t-T_{I}\right)}+\frac{A_{B}}{\mu_{B}^{2}}-\frac{A_{G}}{\mu_{B}}+\frac{A_{G}}{\mu_{G}} \end{matrix}\right]\right\}\right|_{I=A,V} \\ +\frac{F_{A}+F_{V}}{F_{A}+F_{V}+K_{I}}\left.\left\{F_{I}\left[\begin{matrix} +\frac{A_{B}T_{E}}{1-\mu_{B}T_{E}}\left(t-T_{I}\right)e^{-\mu_{B} \left(t-T_{I}\right)} \\ -\frac{T_{E}}{1-\mu_{B}T_{E}}\left(\frac{A_{B}T_{E}}{1-\mu_{B}T_{E}}+A_{G} \right)e^{-\mu_{B} \left(t-T_{I}\right)} \\ +\frac{A_{G}T_{E} }{1-\mu_{G}T_{E}}e^{-\mu_{G} \left(t-T_{I}\right)} \\ +T_{E}\left(\frac{A_{G}}{1-\mu_{B}T_{E}}-\frac{A_{G}}{1-\mu_{G}T_{E}}+\frac{A_{B}T_{E}}{\left(1-\mu_{B}T_{E}\right)^{2}}\right)e^{-\frac{t-T_{I}}{T_{E}}} \end{matrix}\right]\right\}\right|_{I=A,V} \end{matrix}$ (8c)

$=\left.\left\{F_{I}\left[\begin{matrix} +\left[-\frac{K_{I}}{F_{A}+F_{V}+K_{I}}\frac{A_{B} }{\mu_{B}}+\frac{F_{A}+F_{V}}{F_{A}+F_{V}+K_{I}}\frac{A_{B}T_{E}}{1-\mu_{B}T_{E}}\right]\left(t-T_{I}\right)e^{-\mu_{B} \left(t-T_{I}\right)} \\ +\left[\frac{K_{I}}{F_{A}+F_{V}+K_{I}}\left(\frac{A_{G} }{\mu_{B}}-\frac{A_{B} }{\mu_{B}^{2}}\right)-\frac{F_{A}+F_{V}}{F_{A}+F_{V}+K_{I}}\frac{T_{E}}{1-\mu_{B}T_{E}}\left(\frac{A_{B}T_{E}}{1-\mu_{B}T_{E}}+A_{G} \right)\right]e^{-\mu_{B} \left(t-T_{I}\right)} \\ +\left[-\frac{K_{I}}{F_{A}+F_{V}+K_{I}}\frac{A_{G} }{\mu_{G}}+\frac{F_{A}+F_{V}}{F_{A}+F_{V}+K_{I}}\frac{A_{G}T_{E} }{1-\mu_{G}T_{E}}\right]e^{-\mu_{G} \left(t-T_{I}\right)} \\ +\frac{K_{I}}{F_{A}+F_{V}+K_{I}}\left(\frac{A_{B}}{\mu_{B}^{2}}-\frac{A_{G}}{\mu_{B}}+\frac{A_{G}}{\mu_{G}}\right) \\ +\frac{F_{A}+F_{V}}{F_{A}+F_{V}+K_{I}}T_{E}\left(\frac{A_{G}}{1-\mu_{B}T_{E}}-\frac{A_{G}}{1-\mu_{G}T_{E}}+\frac{A_{B}T_{E}}{\left(1-\mu_{B}T_{E}\right)^{2}}\right)e^{-\frac{t-T_{I}}{T_{E}}} \end{matrix}\right]\right\}\right|_{I=A,V}$ (8d)

$=\left.\left\{F_{I}\left[\begin{matrix} +\left[-\frac{K_{I}}{F_{A}+F_{V}+K_{I}}\frac{A_{B} }{\mu_{B}}+\frac{F_{A}+F_{V}}{F_{A}+F_{V}+K_{I}}\frac{A_{B}}{1/T_{E}-\mu_{B}}\right]\left(t-T_{I}\right)e^{-\mu_{B} \left(t-T_{I}\right)} \\ +\left[\frac{K_{I}}{F_{A}+F_{V}+K_{I}}\left(\frac{A_{G} }{\mu_{B}}-\frac{A_{B} }{\mu_{B}^{2}}\right)-\frac{F_{A}+F_{V}}{F_{A}+F_{V}+K_{I}}\frac{1}{1/T_{E}-\mu_{B}}\left(\frac{A_{B}}{1/T_{E}-\mu_{B}}+A_{G} \right)\right]e^{-\mu_{B} \left(t-T_{I}\right)} \\ +\left[-\frac{K_{I}}{F_{A}+F_{V}+K_{I}}\frac{A_{G} }{\mu_{G}}+\frac{F_{A}+F_{V}}{F_{A}+F_{V}+K_{I}}\frac{A_{G} }{1/T_{E}-\mu_{G}}\right]e^{-\mu_{G} \left(t-T_{I}\right)} \\ +\frac{K_{I}}{F_{A}+F_{V}+K_{I}}\left(\frac{A_{B}}{\mu_{B}^{2}}-\frac{A_{G}}{\mu_{B}}+\frac{A_{G}}{\mu_{G}}\right) \\ +\frac{F_{A}+F_{V}}{F_{A}+F_{V}+K_{I}}\left(\frac{A_{G}}{1/T_{E}-\mu_{B}}-\frac{A_{G}}{1/T_{E}-\mu_{G}}+\frac{A_{B}}{\left(1/T_{E}-\mu_{B}\right)^{2}}\right)e^{-\frac{F_{A}+F_{V}+K_{I}}{V_{E}}\left(t-T_{I}\right)} \end{matrix}\right]\right\}\right|_{I=A,V}$ a(8e)

$=\left.\left\{F_{I}\left[\begin{matrix} +\left[-\frac{K_{I}}{F_{A}+F_{V}+K_{I}}\frac{A_{B} }{\mu_{B}}+\frac{F_{A}+F_{V}}{F_{A}+F_{V}+K_{I}}\frac{A_{B}V_{E}}{F_{A}+F_{V}+K_{I}-\mu_{B}V_{E}}\right]\left(t-T_{I}\right)e^{-\mu_{B} \left(t-T_{I}\right)} \\ +\left[\begin{matrix} \frac{K_{I}}{F_{A}+F_{V}+K_{I}}\left(\frac{A_{G} }{\mu_{B}}-\frac{A_{B} }{\mu_{B}^{2}}\right) \\ -\frac{F_{A}+F_{V}}{F_{A}+F_{V}+K_{I}}\frac{V_{E}}{F_{A}+F_{V}+K_{I}-\mu_{B}V_{E}}\left(\frac{A_{B}V_{E}}{F_{A}+F_{V}+K_{I}-\mu_{B}V_{E}}+A_{G} \right) \end{matrix}\right]e^{-\mu_{B} \left(t-T_{I}\right)} \\ +\left[-\frac{K_{I}}{F_{A}+F_{V}+K_{I}}\frac{A_{G} }{\mu_{G}}+\frac{F_{A}+F_{V}}{F_{A}+F_{V}+K_{I}}\frac{A_{G} V_{E}}{F_{A}+F_{V}+K_{I}-\mu_{G}V_{E}}\right]e^{-\mu_{G} \left(t-T_{I}\right)} \\ +\frac{K_{I}}{F_{A}+F_{V}+K_{I}}\left(\frac{A_{B}}{\mu_{B}^{2}}-\frac{A_{G}}{\mu_{B}}+\frac{A_{G}}{\mu_{G}}\right) \\ +\frac{F_{A}+F_{V}}{F_{A}+F_{V}+K_{I}}V_{E}\left(\begin{matrix} \frac{A_{G}}{F_{A}+F_{V}+K_{I}-\mu_{B}V_{E}}-\frac{A_{G}}{F_{A}+F_{V}+K_{I}-\mu_{G}V_{E}} \\ +\frac{A_{B}V_{E}}{\left(F_{A}+F_{V}+K_{I}-\mu_{B}V_{E}\right)^{2}} \end{matrix}\right)e^{-\frac{F_{A}+F_{V}+K_{I}}{V_{E}}\left(t-T_{I}\right)} \end{matrix}\right]\right\}\right|_{I=A,V}$ (8f)

$=\left.\left\{F_{I}\left[\begin{matrix} +A_{B} \frac{\mu_{B}V_{E}-K_{I}}{\mu_{B}\left(F_{A}+F_{V}+K_{I}-\mu_{B}V_{E}\right)}\left(t-T_{I}\right)e^{-\mu_{B} \left(t-T_{I}\right)} \\ -\left[\begin{matrix} +A_{B}\frac{\left(\mu_{B}V_{E}-K_{I}\right)^{2}+\left(F_{A}+F_{V}\right)K_{I}}{\mu_{B}^{2}\left(F_{A}+F_{V}+K_{I}-\mu_{B}V_{E}\right)^{2}} \\ +A_{G} \frac{\mu_{B}V_{E}-K_{I}}{\mu_{B}\left(F_{A}+F_{V}+K_{I}-\mu_{B}V_{E}\right)} \end{matrix}\right]e^{-\mu_{B} \left(t-T_{I}\right)} \\ +A_{G} \frac{\mu_{G}V_{E}-K_{I}}{\mu_{G}\left(F_{A}+F_{V}+K_{I}-\mu_{G}V_{E}\right)}e^{-\mu_{G} \left(t-T_{I}\right)} \\ +\frac{F_{A}+F_{V}}{F_{A}+F_{V}+K_{I}}V_{E}^{2}\left(\begin{matrix} +A_{B}\frac{1}{\left(F_{A}+F_{V}+K_{I}-\mu_{B}V_{E}\right)^{2}} \\ +A_{G}\frac{\left(\mu_{B}-\mu_{G}\right)}{\left(F_{A}+F_{V}+K_{I}-\mu_{B}V_{E}\right)\left(F_{A}+F_{V}+K_{I}-\mu_{G}V_{E}\right)} \end{matrix}\right)e^{-\frac{F_{A}+F_{V}+K_{I}}{V_{E}}\left(t-T_{I}\right)} \\ +\frac{K_{I}}{F_{A}+F_{V}+K_{I}}\left(\frac{A_{B}}{\mu_{B}^{2}}-\frac{A_{G}}{\mu_{B}}+\frac{A_{G}}{\mu_{G}}\right) \end{matrix}\right]\right\}\right|_{I=A,V}.$ (8g)
