# Supplementary material for: A pharmacokinetic model including arrival time for two inputs and compensating for varying applied flip-angle in dynamic gadoxetic acid-enhanced MR imaging
Source: PLoS One. 2019 Aug 15;14(8):e0220835. doi: 10.1371/journal.pone.0220835 (PMC6695151; doi:10.1371/journal.pone.0220835)
Supplement: S3 Appendix — (DOCX) [file pone.0220835.s003.docx]

**S3 Appendix. Derivation from signal intensity to tissue concentration**

In DCE-MRI, the signal in steady state is given by (neglecting T_2_^*^ effect):

$$S\left(T_{1}\right)=N\left(H\right)\sin\left(\alpha\right)\frac{1-e^{-\frac{TR}{T_{1}}}}{1-\cos\left(\alpha\right)e^{-\frac{TR}{T_{1}}}},$$

where N(H) is the local proton density multiplied by an arbitrary factor, *T*_1_ the spin-lattice relaxation time, α the flip-angle and TR the repetition time.

The Relative Signal Intensity (RSI) in a voxel can be calculated as

$$\begin{matrix} RSI\left(T_{1}\right)=\frac{S\left(T_{1}\right)}{S\left(T_{10}\right)} \\ =\frac{1-e^{-\frac{TR}{T_{1}}}}{1-\cos\left(\alpha\right)e^{-\frac{TR}{T_{1}}}}\frac{1-\cos\left(\alpha\right)e^{-\frac{TR}{T_{10}}}}{1-e^{-\frac{TR}{T_{10}}}} \\ =\frac{1-E}{1-\cos\left(\alpha\right)E}F, \end{matrix}$$

in which

$$\left\{\begin{matrix} E=e^{-\frac{TR}{T_{1}}} \\ F=\frac{1-\cos\left(\alpha\right)e^{-\frac{TR}{T_{10}}}}{1-e^{-\frac{TR}{T_{10}}}} \end{matrix}\right..$$

Solving for E yields

$$E=\frac{F-RSI\left(T_{1}\right)}{F-\cos\left(\alpha\right)RSI\left(T_{1}\right)}.$$

Expanding *E* and solving for 1/T_1_(t):

$$\frac{1}{T_{1}}=-\frac{1}{TR}\ln\left(\frac{F-RSI\left(T_{1}\right)}{F-\cos\left(\alpha\right)RSI\left(T_{1}\right)}\right).$$

Next, the concentration of contrast agent can be computed from T_1_:

$$\frac{1}{T_{1}\left(t\right)}=\frac{1}{T_{0}}+RC_{t}\left(t\right),$$

with R the relaxivity of the applied contrast agent.

Solving *C*_T_ (t):

$$\begin{matrix} C_{T}\left(t\right)=\frac{1}{R}\left(\frac{1}{T_{1}\left(t\right)}-\frac{1}{T_{10}}\right) \\ =\frac{1}{R}\left(-\frac{1}{TR}\ln\left(\frac{F-RSI\left(T_{1}\right)}{F-\cos\left(\alpha\right)RSI\left(T_{1}\right)}\right)-\frac{1}{T_{10}}\right) \\ =\frac{1}{R}\left(-\frac{1}{TR}\ln\left(\frac{\frac{1-\cos\left(\alpha\right)e^{-\frac{TR}{T_{10}}}}{1-e^{-\frac{TR}{T_{10}}}}-RSI\left(T_{1}\right)}{\frac{1-\cos\left(\alpha\right)e^{-\frac{TR}{T_{10}}}}{1-e^{-\frac{TR}{T_{10}}}}-\cos\left(\alpha\right)RSI\left(T_{1}\right)}\right)-\frac{1}{T_{10}}\right) \end{matrix}$$

which is as same as Eq (13) in the manuscript.
