# Supplementary material for: A pharmacokinetic model including arrival time for two inputs and compensating for varying applied flip-angle in dynamic gadoxetic acid-enhanced MR imaging
Source: PLoS One. 2019 Aug 15;14(8):e0220835. doi: 10.1371/journal.pone.0220835 (PMC6695151; doi:10.1371/journal.pone.0220835)
Supplement: S4 Appendix — (DOCX) [file pone.0220835.s004.docx]

**S4 Appendix. Fitting results of input function models**

Table 1 Estimated parameters, root mean square error (rmse) and coefficient of determination (R^2^) expressing the goodness of fit of Orton’s model for the liver’s AIF in each patient

| **Case** | **Estimated parameters** | | | | | **Goodness of fit** | |
| --- | --- | --- | --- | --- | --- | --- | --- |
|  | **aB** | **mB** | **aG** | **mG** | **t0** | **rmse** | **R^2^** |
| **1** | 36.1 | 0.264 | 0.015 | 0.00202 | 35.9 | 0.0661 | 0.989 |
| **2** | 58.7 | 0.410 | 0.00839 | 0.00171 | 35.9 | 0.158 | 0.984 |
| **3** | 48.3 | 0.376 | 0.0166 | 0.00223 | 35.3 | 0.247 | 0.941 |
| **4** | 42.1 | 0.129 | 0.0116 | 0.00192 | 35.9 | 0.162 | 0.902 |
| **5** | 26.5 | 0.248 | 0.0196 | 0.00252 | 40.1 | 0.0823 | 0.968 |
| **6** | 22.6 | 0.457 | 0.033 | 0.00563 | 33.9 | 0.0747 | 0.982 |
| **7** | 7.82 | 0.232 | 0.0227 | 0.00245 | 39.4 | 0.032 | 0.947 |
| **8** | 52.7 | 0.185 | 0.00937 | 0.00161 | 38.4 | 0.249 | 0.896 |
| **9** | 35.6 | 0.677 | 0.0141 | 0.00242 | 35.9 | 0.0915 | 0.991 |
| **10** | 55.7 | 0.513 | 0.0136 | 0.0041 | 42.4 | 0.307 | 0.947 |
| **11** | 27.1 | 0.453 | 0.0262 | 0.00513 | 37.6 | 0.168 | 0.937 |
| **Overall** | 37.6 | 0.359 | 0.0173 | 0.00288 | 37.3 | 0.149 | 0.953 |

Table 2 Estimated parameters, root mean square error (rmse) and coefficient of determination (R^2^) expressing the goodness of fit of Orton’s model for the liver’s VIF in each patient.

| **Case** | **Estimated parameters** | | | | | **Goodness of fit** | |
| --- | --- | --- | --- | --- | --- | --- | --- |
|  | **aB** | **mB** | **aG** | **mG** | **t0** | **rmse** | **R^2^** |
| **1** | 20.1 | 0.0797 | 0.0161 | 0.00141 | 45.9 | 0.0513 | 0.980 |
| **2** | 28.4 | 0.038 | 0.00938 | 0.00100 | 43.7 | 0.0708 | 0.898 |
| **3** | 3.78 | 0.312 | 0.0569 | 0.00100 | 44.8 | 0.0669 | 0.933 |
| **4** | 52.9 | 0.0341 | 0.00491 | 0.00100 | 52.6 | 0.144 | 0.859 |
| **5** | 12.9 | 0.111 | 0.0229 | 0.00227 | 51.2 | 0.0415 | 0.985 |
| **6** | 21.7 | 0.0656 | 0.0148 | 0.00127 | 39.3 | 0.0614 | 0.960 |
| **7** | 10.3 | 0.128 | 0.0239 | 0.00194 | 50.7 | 0.0436 | 0.979 |
| **8** | 24.3 | 0.0929 | 0.0176 | 0.00149 | 46.8 | 0.0533 | 0.989 |
| **9** | 11.4 | 0.111 | 0.0206 | 0.00137 | 41.5 | 0.0319 | 0.987 |
| **10** | 15.8 | 0.0607 | 0.0155 | 0.00100 | 49.9 | 0.0371 | 0.970 |
| **11** | 10.7 | 0.202 | 0.0333 | 0.00100 | 42.3 | 0.109 | 0.947 |
| **Overall** | 19.3 | 0.112 | 0.0214 | 0.00134 | 46.2 | 0.0646 | 0.954 |
